# Medication Adherence in Palliative Care Patients

**DOI:** 10.7759/cureus.25322

**Published:** 2022-05-25

**Authors:** Waleed AlShehri, Mohammed Almotairi, Hasan Alshakhs, Razan Orfali

**Affiliations:** 1 Pharmacy, King Fahad Medical City, Riyadh, SAU; 2 Clinical Pharmacy Department, King Fahad Medical City, Riyadh, SAU; 3 Inpatient Pharmacy, King Fahad Medical City, Riyadh, SAU; 4 Research Center, Biomedical Administration, Inpatient Pharmacy, King Fahad Medical City, Riyadh, SAU

**Keywords:** adherence to therapy, outpatient clinic, cancer and oncology, medication adherence strategies, palliative care services

## Abstract

Background

In palliative care, therapeutic benefit and desired health outcome might be affected by non-adherence to medications, especially among patients with advanced illnesses, such as cancer. The consequences of non-adherence to medications could include poor health outcomes, recurrent admissions, medication waste, as well as increased morbidity and mortality. The aim of this study was to measure the level of medication adherence in palliative care patients visiting the outpatient clinic at King Fahad Medical City.

Methods

Inclusion criteria comprised all palliative care patients visiting the outpatient clinic in King Fahad Medical City. Medication adherence was assessed among the participants using the Morisky Medication Adherence Scale (MMAS). Data analysis was conducted using SPSS and GraphPad Prism.

Results

A total of 84 responses were recorded. Among the respondents, 58.3% were female. The most common underlying diseases among participants were breast cancer. Of the 84 participants, 59 (70.2%) patients reported good adherence, while 25 (29.7%) reported poor adherence.

Conclusion

Non-adherence to medications among palliative care patients is a significant public health problem. Results indicated that the overall level of medication adherence in palliative care patients was moderate to good. Further studies are required to design new techniques for increasing medication adherence in palliative care patients.

## Introduction

An estimated 20% to 50% of patients do not adhere to their prescribed treatment regimens and are said to be “non-compliant” and “non-adherent” with therapy [1-3]. Medication adherence (MA) is an essential matter for achieving the goals of treatment. Adherence could highly influence the effectiveness of these medications [4-6], especially with patients who have advancing problems such as cancer [7-8]. Poor health outcomes and potential morbidity and mortality could be consequences of non-adherence to medications [9-10].

There is limited research on adherence to medications by palliative care patients towards the end of life [4]. Various factors such as age, gender, adverse events, comorbidities, concomitant medications, dosing frequencies, and capacity to perform tasks have been shown to influence adherence and persistence [1,5]. However, decisions involving medication management at the end of life are complicated by many factors, including the nature and severity of the disease, the patient’s age, the efficacy of life-sustaining treatment, and the emotions and values of the patient and their family [4,6].

Palliative care clinics aim to enhance the quality of life for both the patient and their families by addressing uncomfortable symptoms, such as pain, nausea, vomiting, dyspnoea, and constipation [7]. Palliative care also intensifies and focuses on aggressive symptom management regardless of diagnosis to avoid unnecessary emergencies and hospital stays [7-8]. Ongoing medications are usually a basic part of a palliative care treatment plan for the management of symptoms. Clinical pharmacists can play a vital role in palliative care in several ways, including assessment of medication plans and informing patients about rational drug use. Ongoing medications are usually a core part of a palliative care treatment plan for the management of symptoms. Thus, the involvement of clinical pharmacists is necessary, especially when therapies involve medications with adverse effects that can compromise treatment adherence [2-5].

Adherence research in palliative care has mainly focused on quantifying the problem, except for a few studies [9-10] that have attempted to identify the factors influencing non-adherence. Although some literature deals with end-of-life care in palliative care patients, only a few studies have focused on the medication use patterns. Measuring medication adherence and exploring factors and reasons for non-adherence might help understand any potential deficiency in health care and medication use practice towards the palliative care and better inform patient management [11].

The overall aim of this study was to address medication adherence in palliative care patients visiting the outpatient clinic in King Fahad Medical City (KFMC), Riyadh. The primary objective of this study was to find out the reasons behind non-adherence to medications so that we could identify the proper solutions to minimize or even eliminate any obstacle that might lead to non-adherence.

## Materials and methods

A prospective observational study of palliative care patients with different types of cancer in King Fahad Medical City was conducted using KFMC outpatient files. Data collection was made using a modified Morisky Medication Adherence Scale (MMAS), including the original four questions of the MMAS 4-item scale. Plus, one additional question to address the persistence of taking medication [12]. The written self-report questionnaires (translated into Arabic) were collected as part of usual care by a palliative care nurse from almost all palliative care patients during their visits (February 2019 - December 2019). Patients in palliative care clinics are educated by the pharmacist through various methods, including simplifying their medications, identifying reasons for nonadherence, and asking specific questions about their medications.

Cohort identification

Inclusion Criteria

All palliative care patients, males, and females, >1 8-year-old visiting a palliative care outpatient clinic in KFMC either as a first visit or for follow-up were included in this study. Participants who met the inclusion criteria and agreed to participate will either complete if able to read and write the MMAS or be asked verbally by the clinical pharmacist to answer these questions. The term 'comorbidity' refers to when an individual simultaneously has more than one illness.

Measures

Medication Adherence

The MMAS was used to observe the medication adherence of participants. It contains five items regarding medication adherence (record of patient's medication-taking behavior). Each item was rated on a five-point Likert scale, and the range of the MMAS total score is between five and 25. A higher score on the MMAS represents better medication adherence [13-14].

Statistical analysis procedure

Data analysis was conducted using SPSS version 25.0 (SPSS Inc., Chicago, IL, USA) and GraphPad Prism 9. Demographic characteristics of palliative care patients, including all categorical variables such as age group, gender, and type of disease, were presented as numbers and percentages. Chi-square statistics/Fisher's exact tests were used to compare proportions between dichotomous or categorical variables. Descriptive statistics were expressed as mean, standard deviation, and minimum and maximum values. A P-value less than 0.05 is considered statistically significant. The normality of the data was assessed with a histogram, QQ plot, and the Shapiro-Wilk test. Since the data obtained from the scale was normally distributed, the student's t-test was used to compare the scores of the participants. The Chi-square test also was applied to determine the significant association between categorical variables. Finally, Pearson's correlation coefficient was calculated to see whether there was a correlation between patients receiving palliative care and medication adherence.

Ethical approval

This study was approved by the IRB Ethics Committee of the King Fahad Medical City. Informed consent was obtained from each patient verbally. The patients were assured of their right to refuse to participate or withdraw from the study at any stage.

## Results

Sample characteristics

Table 1 represents the participants' demographic information, clinical characteristics, and medication adherence (N = 84). Major themes were pertaining to the use of medications: modification of daily dose, discontinuation of medications, and other adherence behaviors that were identified through content and thematic analysis of the detailed interviews. A total of 84 responses were received. There were more female (58.3%) than male patients among the respondents. The most common underlying diseases among participants were breast cancer (26.2%), followed by rectal cancer (10.7%), and other types of cancer (63.1%) reported by the respondents.

**Table 1:** Distribution of demographic (n =84)

Characteristics	Description	n(n%)
Age group	< 40	16 (19.0%)
	40 - 60	31 (36.9%)
	> 60	37 (44.0%)
Gender	Male	35 (41.7%)
	Female	49 (58.3%)
Palliative diagnosis	Breast Cancer	22 (26.2%)
	Rectal Cancer	9 (10.7%)
	Other Cancer	53 (63.1%)
Presence of Comorbidities	With Comorbidities	36 (42.9%)
	Without Comorbidities	48 (57.1%)

Adherence to treatment

Among patients who visited the palliative care outpatient clinic in KFMC, 59 patients (70.2%) reported good adherence, while 25 of the 84 patients (29.8%) reported poor adherence. No significant differences were observed between patients who self-reported good adherence and those who reported poor adherence in their demographic and clinical features (Table 2).

**Table 2:** Statements about use of medicines *5 point Likert scale, always= 1, usually=2, sometimes=3, rare=4, never=5(total 25), Good Adherence >12.5, Poor Adherence <12.5

I forget to take my medications	Always	3 (3.6%)
	Usually	1 (1.2%)
	Sometimes	6 (7.1%)
	Rare	10 (11.9%)
	Never	64 (76.2%)
Sometimes I modify my daily dose	Always	3 (3.6%)
	Usually	3 (3.6%)
	Sometimes	11 (13.1%)
	Rare	8 (9.5%)
	Never	59 (70.2%)
I stop taking my medications for a period of time	Always	2 (2.4%)
	Usually	1 (1.2%)
	Sometimes	10 (11.9%)
	Rare	6 (7.1%)
	Never	65 (77.4%)
I decide not to take a certain dose	Always	3 (3.6%)
	Usually	3 (3.6%)
	Sometimes	7 (8.3%)
	Rare	10 (11.9%)
	Never	61 (72.6%)
I take less than the recommended treatments.	Always	2 (2.4%)
	Usually	0 (0.0%)
	Sometimes	6 (7.1%)
	Rare	9 (10.7%)
	Never	67 (79.8%)

**Figure 1:**
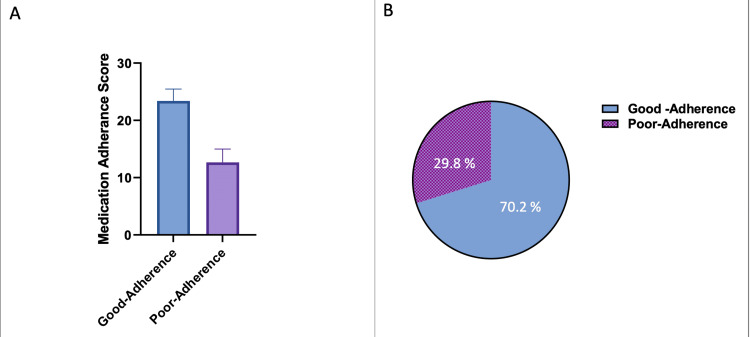
A) mean scores of Good Adherence and Poor Adherence participants, B) The percentage of Medication adherence scores.

The mean index scores of the adherent participants on the Morisky Medication Adherence scale were calculated as 23.31 ± 2.10 for good adherence and 11 ± 3.4 as a poor adherence (Figure 1).

There was a statistically significant difference between males and females on their decision for specific doses in the scale statement “I decide not to take a certain dose” (P < 0.05). (80.0%) of males were rarely decided not to take a certain dose. However, only (20.0%) percent of females rarely decided not to take a certain dose.

## Discussion

Currently, few studies focus on patient adherence to medications in the palliative care setting. In the palliative care practice, the prescriptions for each patient usually include an average of five to six drugs [8,15]. The number of prescribed medications is directly connected to therapeutic adherence: the greater the number of prescribed medications, the more daily drug administration [16-17]. Poor adherence has been associated with a rise in physician visits, greater hospitalization rates, and lengthier hospital stays. Understanding medication adherence in palliative care patients becomes essential. 

Overall, measured adherence to medications in palliative care was good. The findings indicate that issues related to age, gender, type of disease, and comorbidities as measured using MMAS, are well taken care of in the study sample investigated [18-19]. Good adherence may, in part, be explained by considering the characteristics of the settings. Most of the participants studied received care from palliative care pharmacists. They were all identified at specialist health care services where health personnel are likely to be experienced with the treatment of cancer patients [11-12]. Good adherence was observed in both participants, with comorbidities and without comorbidities (Table 1, Figure 1). Among patients with comorbidity, 35 patients (41.7%) reported good adherence. That means comorbidities (The presence of more than one illness in the same individual) did not affect the medication adherence in palliative care patients.

Various reasons for poor adherence have been reported, and these can be classified as non-intentional and intentional poor adherence. Non-intentional poor adherence is often the result of poor memory or understanding [13-14]. As shown in Table 3, with regards to age, there is a statistical significance between the variables regarding the statement “I forget to take my medications. 66.7% of patients < 40 years old always forget to take their medications, while 46.9% of patients > 60 never forget.

**Table 3:** Association between demographic variables and use of medications

Characteristics	Description	I forget to take my Medications					P - value
		Always	Usually	Sometimes	Rare	Never	
Age group	< 40	2 (66.7%)	1 (100.0%)	0 (0.0%)	4 (40.0%)	9 (14.1%)	*0.023
	40 - 60	1 (33.3%)	0 (0.0%)	1 (16.7%)	4 (40.0%)	25 (39 %)	
	> 60	0 (0.0%)	0 (0.0%)	5 (83.3%)	2 (20.0%)	30 (46.9%)	
Gender	Male	1 (33.3%)	1 (100.0%)	2 (33.3%)	4 (40.0%)	27 (42.2%)	0.795
	Female	2 (66.7%)	0 (0.0%)	4 (66.7%)	6 (60.0%)	37 (57.8%)	
Type of disease	Breast Cancer	1 (33.3%)	0 (0.0%)	0 (0.0%)	3 (30.0%)	18 (28.1%)	0.562
	Rectal Cancer	0 (0.0%)	0 (0.0%)	2 (33.3%)	0 (0.0%)	7 (10.9%)	
	Other Cancer	2 (66.7%)	1 (100.0%)	4 (66.7%)	7 (70.0%)	39 (61%)	
Comorbidities	With Comorbidities	1 (33.3%)	0 (0.0%)	3 (50.0%)	3 (30.0%)	29 (45.3%)	0.769
	Without Comorbidities	2 (66.7%)	1 (100.0%)	3 (50.0%)	7 (70.0%)	35 (54.7%)	

The reasons for intentional poor adherence are less straightforward. For example, the statement “Sometimes I modify my daily Dose” and “I stop taking my medications for some time” could include problems experienced as a direct result of taking medicines (such as adverse drug reactions) or the inability to pay for medicines. Our findings demonstrate no statistical significance between almost all variables and intentional modification of daily dose (Table 4). Similarly, there was no statistical significance between demographic variables and discontinuation of medications (Table 5). In addition, there was no correlation between medication adherence score, gender, and age (r = 0.080).

**Table 4:** Association between demographic variables and modification of daily dose

Characteristics	Description	Sometimes I modify my daily dose					P - value
		Always	Usually	Sometimes	Rare	Never	
Age group	< 40	2 (66.7%)	1 (33.3%)	0 (0.0%)	0 (0.0%)	13 (22.0%)	0.143
	40 - 60	1 (33.3%)	0 (0.0%)	6 (54.5%)	3 (37.5%)	21 (35.6%)	
	> 60	0 (0.0%)	2 (66.7%)	5 (45.5%)	5 (62.5%)	25 (42.4%)	
Gender	Male	1 (33.3%)	0 (0.0%)	5 (45.5%)	3 (37.5%)	26 (44.1%)	0.646
	Female	2 (66.7%)	3 (100.0%)	6 (54.5%)	5 (62.5%)	33 (55.9%)	
Type of Disease	Breast Cancer	1 (33.3%)	2 (66.7%)	4 (36.4%)	2 (25.0%)	13 (22.0%)	0.653
	Rectal Cancer	0 (0.0%)	0 (0.0%)	1 (9.1%)	2 (25.0%)	6 (10.2%)	
	Other Cancer	2 (66.7%)	1 (33.3%)	6 (54.5%)	4 (50.0%)	40 (67.8%)	
Comorbidities	With Comorbidities	1 (33.3%)	2 (66.7%)	5 (45.5%)	4 (50.0%)	24 (40.7%)	0.892
	Without Comorbidities	2 (66.7%)	1 (33.3%)	6 (54.5%)	4 (50.0%)	35 (59.3%)	

**Table 5:** Association between demographic variables and discontinuation of medications

Characteristics	Description	I stop taking my medications for a period of time					P - value
		Always	Usually	Sometimes	Rare	Never	
Age group	< 40	2 (66.7%)	1 (50.0%)	1 (10.0%)	0 (0.0%)	12 (19.0%)	0.061
	40 - 60	1 (33.3%)	1 (50.0%)	4 (40.0%)	2 (33.3%)	24 (38.1%)	
	> 60	0 (0.0%)	0 (0.0%)	5 (50.0%)	4 (66.7%)	27 (42.9%)	
Gender	Male	1 (50.0%)	2 (66.7%)	3 (30.0%)	4 (66.7%)	26 (41.3%)	0.578
	Female	1 (50.0%)	1 (33.3%)	7 (70.0%)	2 (33.3%)	37 (58.7%)	
Type of Disease	Breast Cancer	1 (50.0%)	1 (50.0%)	2 (20.0%)	1 (14.3%)	16 (25.4%)	0.837
	Rectal Cancer	0 (0.0%)	0 (0.0%)	1 (10.0%)	1 (14.3%)	7 (11.1%)	
	Other Cancer	1 (50.0%)	1 (50.0%)	7 (70.0%)	5 (71.4%)	40 (63.5%)	
Comorbidities	With Comorbidities	0 (0.0%)	1 (100.0%)	5 (45.5%)	5 (83.3%)	25 (39.7%)	0.128
	Without Comorbidities	2 (100.0%)	0 (0.0%)	6 (54.5%)	2 (16.7%)	38 (60.3%)	

The appropriate medical practice involves decreasing the number of medications used [15]. In order to help patients, adhere, attention has to be given to the frequency of dosing, the total number of tablets a patient is taking, and their adherence level [16]. Long-acting medications that allow once-daily administration is helpful. Awareness of other therapies taken by the same patient is also essential. More medications might affect the patient’s decision for specific doses [17]. A statistically significant difference was found between males and females and the decision for a specific dose (medication adherence) (P < 0.05) (Table 6).

**Table 6:** Association between demographic variables and decision for certain dose

Characteristics	Description	I decide not to take a certain dose					P - value
		Always	Usually	Sometimes	Rare	Never	
Age group	< 40	2 (66.7%)	1 (33.3%)	0 (0.0%)	1 (10.0%)	12 (19.7%)	0.389
	40 - 60	0 (0.0%)	1 (33.3%)	4 (57.1%)	3 (30.0%)	23 (37.7%)	
	> 60	1 (33.3%)	1 (33.3%)	3 (42.9%)	6 (60.0%)	26 (42.6%)	
Gender	Male	2 (66.7%)	0 (0.0%)	3 (42.9%)	8 (80.0%)	22 (36.1%)	*0.045
	Female	1 (33.3%)	3 (100.0%)	4 (57.1%)	2 (20.0%)	39 (63.9%)	
Type of disease	Breast Cancer	1 (33.3%)	1 (33.3%)	2 (28.6%)	1 (10.0%)	17 (27.9%)	0.940
	Rectal Cancer	0 (0.0%)	0 (0.0%)	1 (14.3%)	2 (20.0%)	6 (9.8%)	
	Other Cancer	2 (66.7%)	2 (66.7%)	4 (57.1%)	7 (70.0%)	38 (62.3%)	
Comorbidities	With Comorbidities	1 (33.3%)	3 (100.0%)	3 (42.9%)	6 (60.0%)	23 (37.7%)	0.201
	Without Comorbidities	2 (66.7%)	0 (0.0%)	4 (57.1%)	4 (40.0%)	38 (62.3%)	

No significant differences were observed between demographic variables and decisions for recommended treatment (Table 7). Despite this study's strengths, the number of medications taken is not collected in this study, which is one of the main limitations. Patient adherence is associated with a patient choice [1], consistent with the palliative goal of patient-centered care. However, this study did not detect if adherence was high because the patient intended to adhere (versus family pressure to adhere). In-depth consideration of the reasons for the high adherence in palliative care patients would help provide insight into this phenomenon, and understand how patient choice affects the level of adherence [15,17].

**Table 7:** Association between demographic variables and decision for recommended treatment

Characteristics	Description	I take less than the recommended treatments.					P - value
		Always	Usually	Sometimes	Rare	Never	
Age group	< 40	2 (100.0%)	0 (0.0%)	1 (16.7%)	1 (11.1%)	12 (17.9%)	0.107
	40 - 60	0 (0.0%)	0 (0.0%)	1 (16.7%)	4 (44.4%)	26 (38.8%)	
	> 60	0 (0.0%)	0 (0.0%)	4 (66.7%)	4 (44.4%)	29 (43.3%)	
Gender	Male	1 (50.0%)	0 (0.0%)	2 (33.3%)	5 (55.6%)	27 (40.3%)	0.803
	Female	1 (50.0%)	0 (0.0%)	4 (66.7%)	4 (44.4%)	40 (59.7%)	
Type of disease	Breast Cancer	1 (50.0%)	0 (0.0%)	2 (33.3%)	3 (33.3%)	16 (23.9%)	0.949
	Rectal Cancer	0 (0.0%)	0 (0.0%)	1 (16.7%)	1 (11.1%)	7 (10.4%)	
	Other Cancer	1 (50.0%)	0 (0.0%)	3 (50.0%)	5 (55.6%)	44 (65.7%)	
Comorbidities	With Comorbidities	0 (0.0%)	0 (0.0%)	1 (16.7%)	7 (77.8%)	28 (41.8%)	0.053
	Without Comorbidities	2 (100.0%)	0 (0.0%)	5 (83.3%)	2 (22.2%)	39 (58.2%)	

## Conclusions

Adherence to medications among palliative care patients is a challenging process. Results of this study indicated that the overall level of adherence in palliative care patients shows moderate to good medication adherence. Although, patients face a variety of barriers to adherence in the palliative care setting. So, the designated interventions should address these obstacles to improve medication adherence in a palliative care setting. Also, it may be particularly effective to target beliefs and concerns in non-adherence to medication, as this is an essential predictor of adherence. Besides, the interaction with a palliative care clinical pharmacist is critical for understanding the reason for non-adherence and promoting adherence through designing new tools for improving medication adherence.
